# The First Documented Case of Ectrodactyly in Ghana: A Case Report

**DOI:** 10.7759/cureus.107019

**Published:** 2026-04-14

**Authors:** Schambach J Sobbin, Adjoa E Hackman, Frank E Gyamfi, Dennis K Kyeremeh, Joseph D Appiagyei, Fareeda Galley

**Affiliations:** 1 Department of Pediatrics, Holy Family Catholic Hospital, Berekum, GHA; 2 Edinburgh Medical School, University of Edinburgh, Edinburgh, GBR; 3 Department of Surgery, Presbyterian Hospital, Dormaa Ahenkro, GHA; 4 Department of Surgery, Holy Family Catholic Hospital, Berekum, GHA; 5 Pediatric Surgery Unit, Komfo Anokye Teaching Hospital, Kumasi, GHA

**Keywords:** advanced maternal age, congenital abnormalities, ectrodactyly, prenatal diagnosis, split-hand-foot malformation

## Abstract

Ectrodactyly, also called split-hand/foot malformation, is a rare congenital limb defect. We report what seems to be the first documented case of ectrodactyly in Ghana. A term female neonate, delivered by elective cesarean section at 38 weeks and 1 day to a 45-year-old multiparous mother with poorly controlled chronic hypertension and well‑controlled gestational diabetes, was found to have bilateral cleft‑type deformities of both hands and feet. Both the index and middle fingers were absent from the right hand, while the middle finger was absent from the left hand. The second and third toes of the left foot and the second toe of the right foot were also absent. No other systemic malformations or a family history of similar defects were identified. Radiographs confirmed findings consistent with ectrodactyly. The case was classified as non‑syndromic, likely sporadic, and possibly linked to a de novo genetic event associated with advanced maternal age.

This report highlights the need for proper documentation and enhanced surveillance of congenital anomalies in the country.

## Introduction

Ectrodactyly is a rare congenital limb anomaly affecting around one in 18,000 to one in 90,000 live births [1,2]. Ectrodactyly, also referred to as split-hand/foot malformation (SHFM), is characterized by a median cleft of the hands and/or feet due to the absence or underdevelopment of the central digital rays [1]. Ectrodactyly results in the distinctive “lobster-claw” appearance due to deep median clefts, syndactyly, or the absence of the metacarpals, metatarsals, and phalanges [1,3]. It may present as a syndromic complex, such as EEC (ectrodactyly-ectodermal dysplasia-cleft lip/palate) syndrome, or it may present as an isolated (non-syndromic) defect [3,4]. Ectrodactyly may impair hand and foot function. In syndromic cases, associated cleft lip or palate defects may also affect feeding, speech, and language development [5].

We highlight the rarity of the congenital abnormality, its presentation, and diagnostic assessment by reporting a case of an isolated type of ectrodactyly affecting all four limbs in a term newborn without a family history of congenital abnormalities.

This article was previously presented as a poster at the maiden Holy Family Catholic Hospital Berekum Research Symposium, held on November 19, 2025.

## Case presentation

A term female neonate was delivered by elective cesarean section at 38 weeks and 1 day of gestation, with a birth weight of 2.8 kg. She was born to a 45-year-old multiparous mother (para 4 [1D] + 0) with poorly controlled chronic hypertension and well‑controlled gestational diabetes mellitus. Apgar scores were 7/10 and 8/10 at 1 and 5 minutes, respectively, and her vital signs were normal. The neonate was transferred to the neonatal intensive care unit (NICU) for closer observation and a comprehensive neonatal examination after the congenital limb abnormalities were identified at birth.

**Figure 1:**
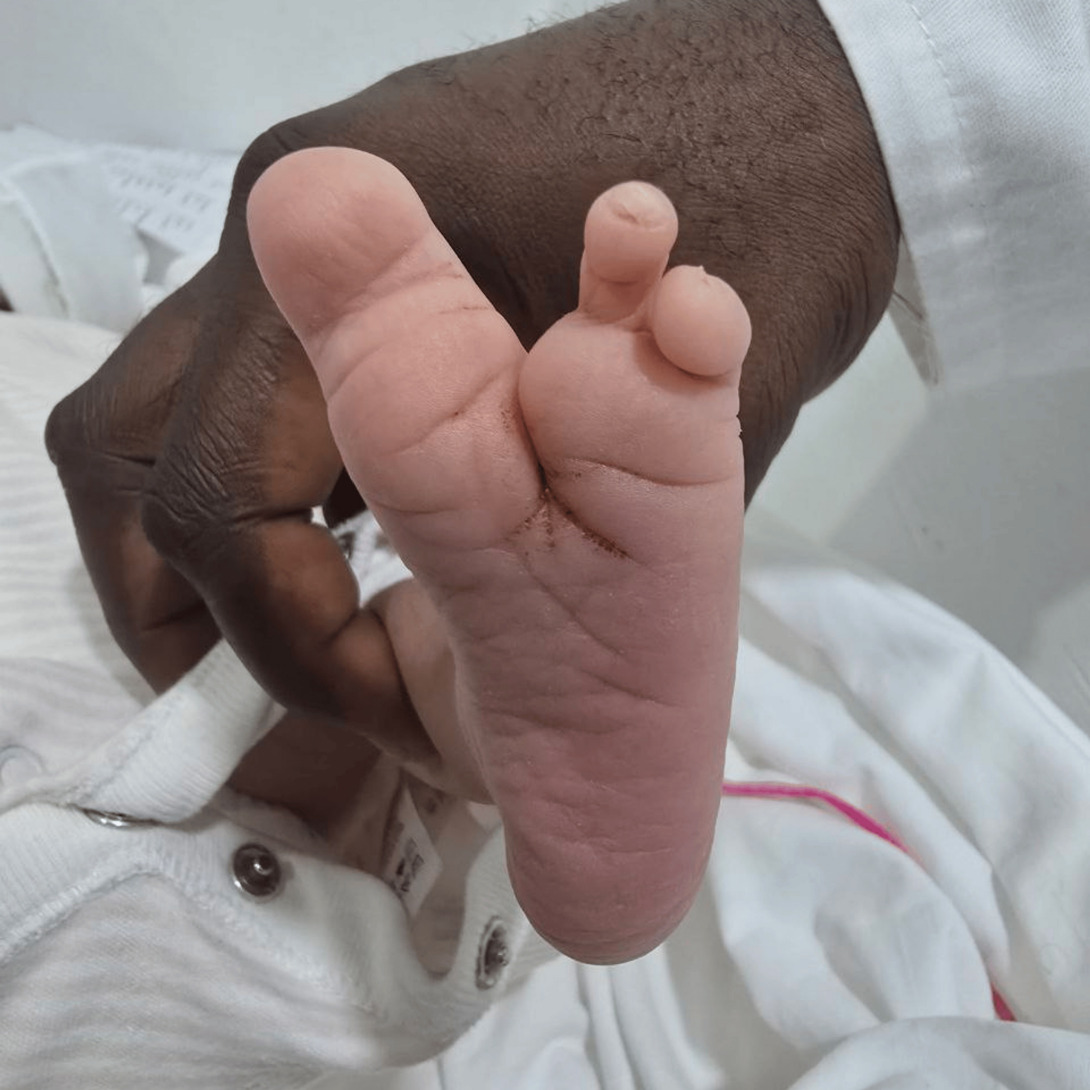
Left foot missing the second and third toes

**Figure 2:**
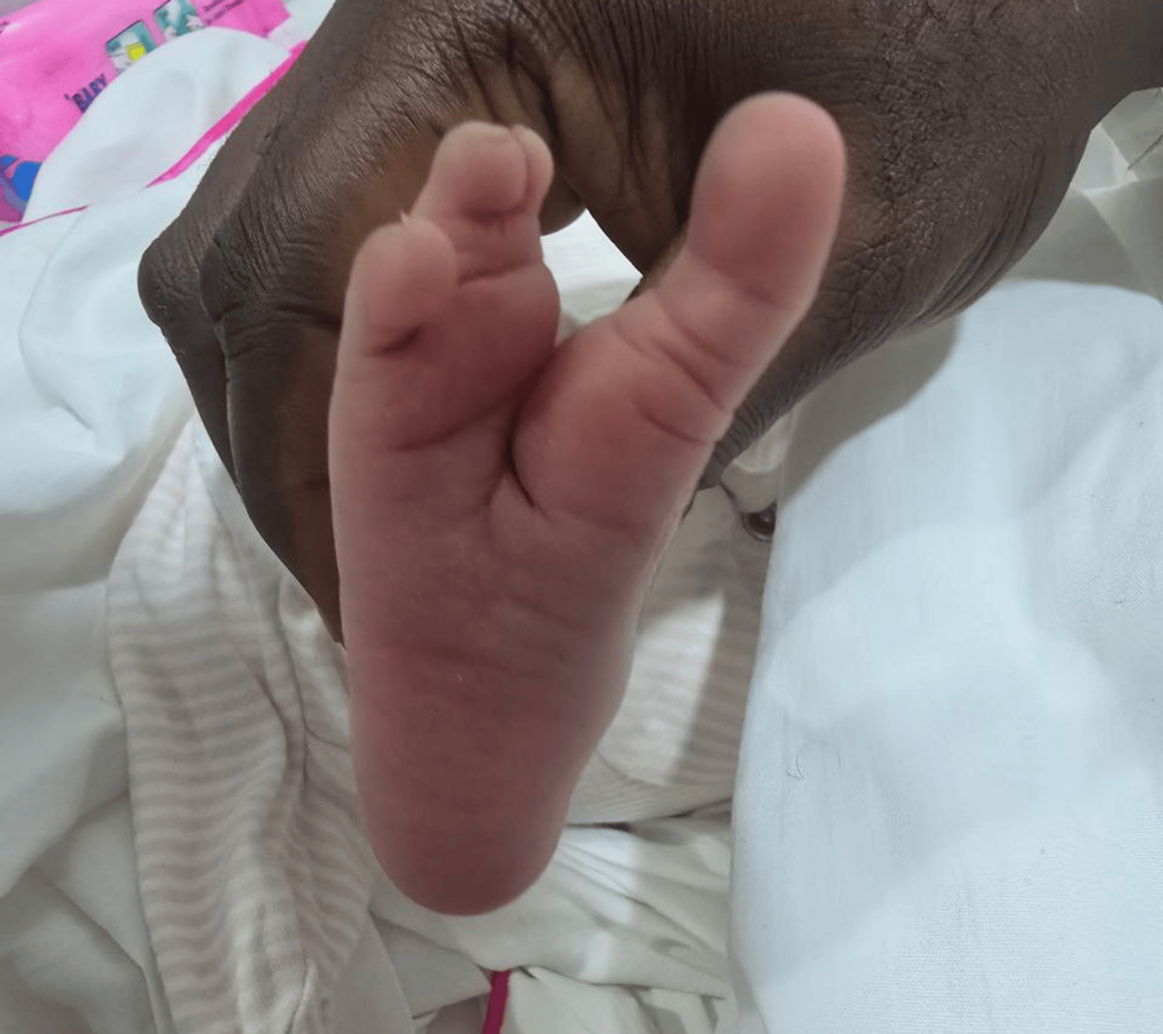
Right foot missing the second toe

**Figure 3:**
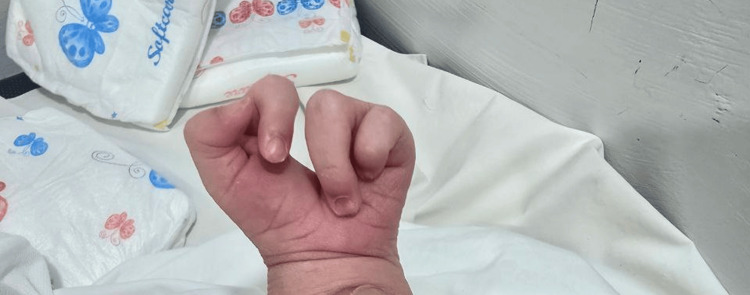
Left hand missing the middle finger

**Figure 4:**
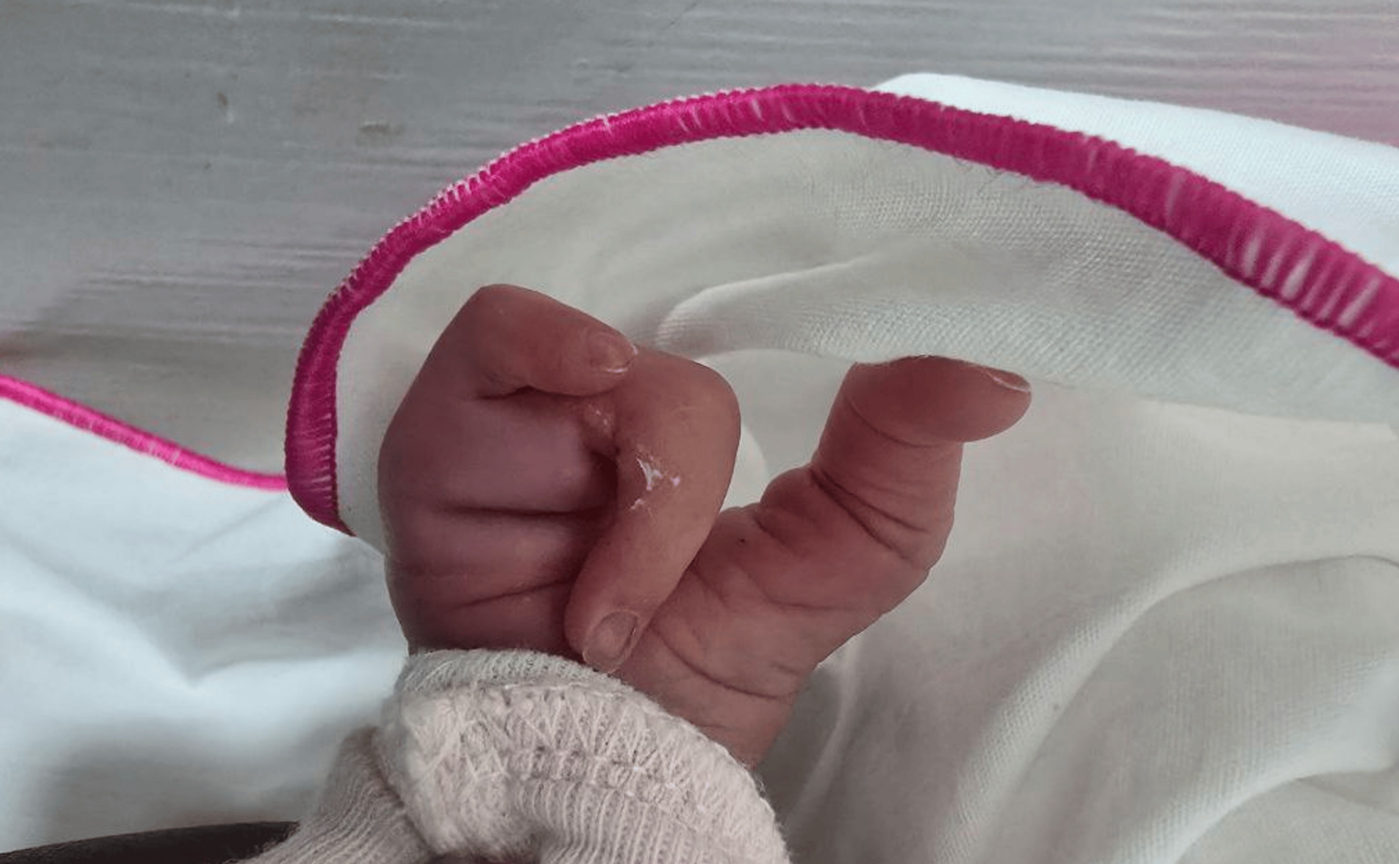
Right hand missing the index and middle fingers

**Figure 5:**
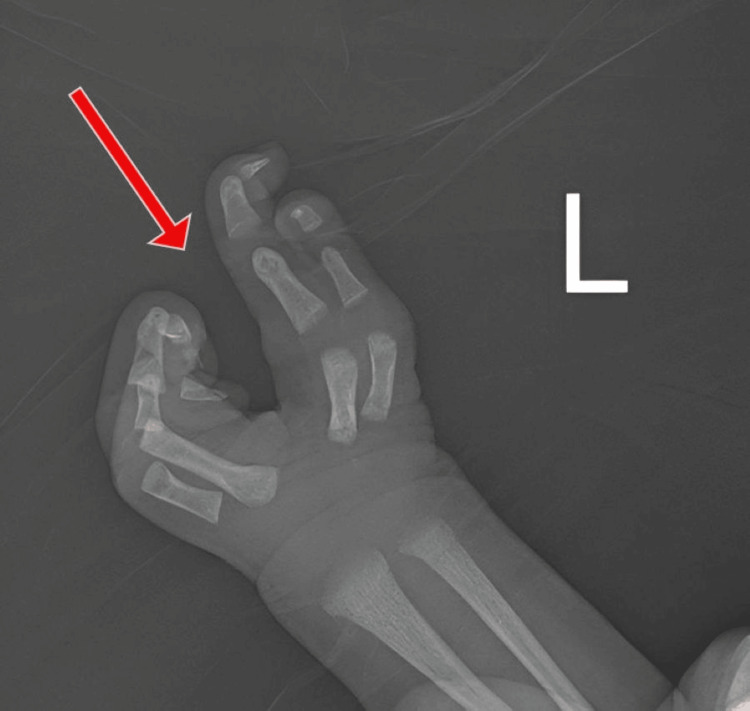
Plain radiograph of the left hand showing the absence of the middle-finger phalanges and metacarpal

**Figure 6:**
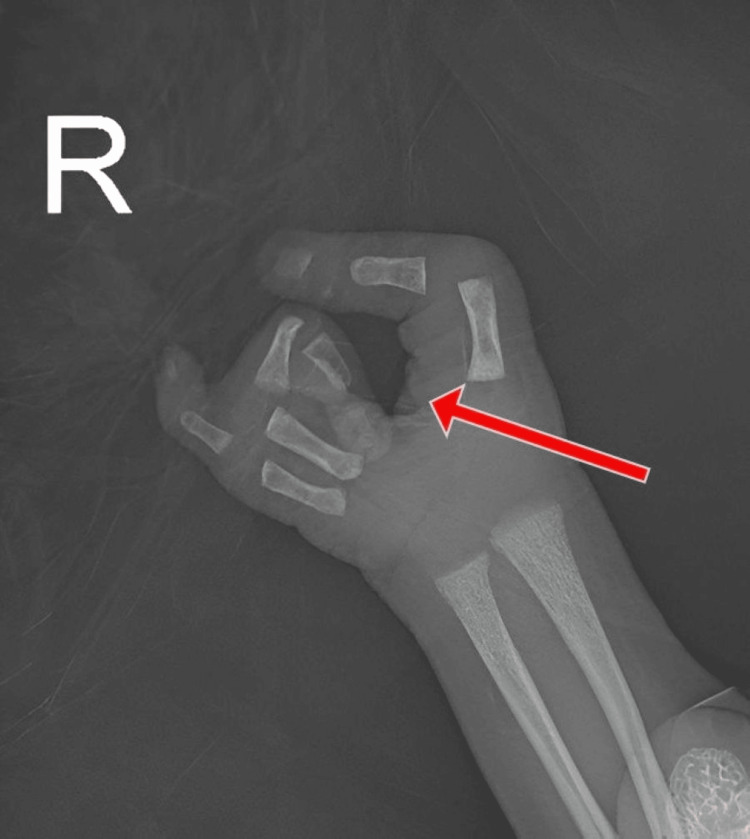
Plain radiograph of the right hand showing the absence of the index- and middle-finger phalanges and metacarpals

**Figure 7:**
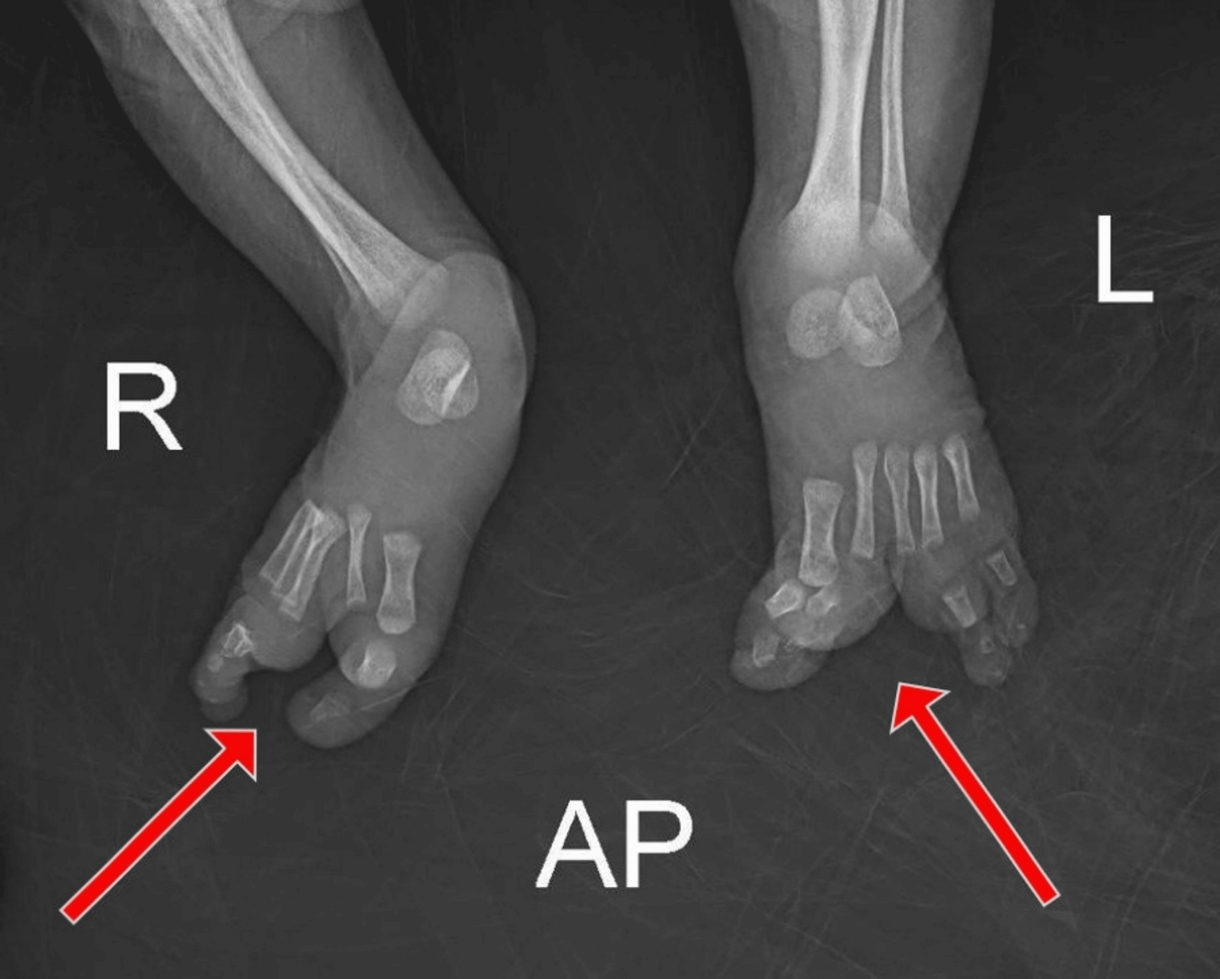
Plain radiograph of both feet showing the left foot missing the phalanges of the second and third toes, and the right foot missing the phalanges of the second toe

The examination showed a claw-shaped deformity in both hands and feet. The second and third toes were missing from the left foot, and the second toe was missing from the right foot, as illustrated in Figure 1 and Figure 2, respectively. The left hand was missing its middle finger, and the right hand was missing both its index and middle fingers, as shown in Figure 3 and Figure 4, respectively. No other congenital abnormalities were identified. There was no family history of limb malformations. Radiographic evaluation of both hands and feet confirmed features consistent with ectrodactyly, as demonstrated in Figures 5-7. Echocardiography was not performed because it was unavailable in our setting. The neonate remained hemodynamically stable, had no neonatal emergencies, and was discharged on day 2 of life for a follow-up after infancy.

The mother booked for antenatal care at 23 weeks and 5 days of gestation. She attended five of the recommended minimum of eight antenatal care contacts. Her chronic hypertension was poorly controlled because of medication non-compliance. Her recorded blood pressures during the pregnancy ranged from 139/83 mmHg to 175/94 mmHg. Her gestational diabetes mellitus was well controlled with diet and exercise therapy, and she did not require pharmacologic treatment. She took ferrous sulfate, folic acid, methyldopa, and nifedipine during pregnancy, with no history of any other medication use. A two‑dimensional fetal anomaly scan performed at 23 weeks 5 days of gestation was reported as normal. 

## Discussion

The reported prevalence of SHFM varies from one in 18,000 to one in 90,000 live births, which accounts for 8-17% of all congenital limb abnormalities [1,2]. Most SHFM cases occur sporadically, as in the present case. Familial forms, though less common, are predominantly inherited in an autosomal dominant fashion, whereas autosomal recessive and X-linked forms have been documented in only a few families [2,6]. Umair and Hayat identified 12 SHFM types mapped to various chromosomal loci. SHFM1 located at 7q21, SHFM2 at Xq32, SHFM3 at 10q24, SHFM4 at 3q27 are some examples. Five key genes found at these loci are DLX5/DLX6, TP63, WNT10B, ZAK, and EPS15L1 [3].

Ectrodactyly may manifest as a syndromic complex, such as EEC syndrome or other SHFM-related syndromes with urogenital or craniofacial abnormalities, or it may manifest as an isolated (non-syndromic) defect [3,4]. The absence of additional anomalies in the current patient suggests an isolated form. TP63 mutations are responsible for about 10-16% of isolated SHFM cases, whereas 93% of EEC syndrome cases have mutations in this gene. Sporadic isolated SHFM cases are likely caused by de novo mutations occurring during early embryogenesis, affecting genes linked to the apical ectodermal ridge (AER) [2]. Ectrodactyly is primarily caused by a disruption in median AER signaling due to increased apoptosis or decreased cell proliferation. The inability of the AER to release signaling molecules required for digital ray formation results in impaired differentiation of the autopod, leading to a split hand or foot appearance [6]. From mild abnormalities, such as hypoplasia of a single phalanx or syndactyly, to severe deformities, such as monodactyly or the classical cleft (lobster-claw) configuration, SHFM exhibits a wide range of phenotypic heterogeneity [2]. Ectrodactyly can be unilateral, can be bilateral, or affect all four limbs [7]. The involvement of both the upper and lower limbs of this neonate is consistent with cases that have been documented in Nigeria [8] and India [6,9]. Table 1 compares the clinical presentation of our patient with that of the Nigerian case reported by Durowaye et al. in 2011 [8].

**Table 1:** Comparison of the present case with a case of familial ectrodactyly reported by Durowaye et al. (2011) in Nigeria

Variable	Present Case	Durowaye et al. (2011) [8]
Country	Ghana	Nigeria
Maternal age	45 years	25 years
Parity	Para 4 (1D)	Para 4 (2D)
Gestational age at antenatal booking	23 weeks	12 weeks
Maternal comorbidities	Chronic hypertension and gestational diabetes mellitus	None reported
Maternal medication use	Ferrous sulfate, folic acid, methyldopa, and nifedipine	Routine antenatal medications only; specific medications not stated
Family history	No family history of limb malformations	Father had ectrodactyly involving the left hand and right foot
Social habits	No smoking, alcohol, or recreational drug use reported	No smoking, alcohol, or recreational drug use reported
Neonatal sex	Female	Male
Birth weight	2.8 kg	3.0 kg
Head circumference	35.0 cm	36.0 cm
Length	46.0 cm	49.5 cm
Limb involvement	Left hand: missing third digit	Left hand: missing third and fourth digits
	Right hand: missing second and third digits	Right hand: missing three central digits
	Left foot: missing second and third digits	Left foot: missing four digits
	Right foot: missing second digit	Right foot: missing four digits
Associated congenital anomalies	None identified on gross examination	Low-set ears
Clinical type	Apparently sporadic, non-syndromic ectrodactyly	Familial, syndromic ectrodactyly syndrome

To the best of our knowledge, ectrodactyly has not been reported before in Ghana. This could be the first-ever documented occurrence in the country. A four-year retrospective study at Korle Bu Teaching Hospital by Swarray-Deen et al. found that congenital abnormalities mostly affected the central nervous, genitourinary, and gastrointestinal systems; limb anomalies were not specifically mentioned [10]. Rather than an actual absence of SHFM, the lack of recorded cases could be due to underdiagnosis, poor reporting, and/or restricted access to genetic testing [11]. This highlights the need for improved case reporting, genetic screening, and publication to enhance Ghana's congenital abnormality surveillance.

Advanced maternal age, as seen in this case (45 years), may contribute to de novo post-zygotic mutations [12]. A systematic review and meta-analysis by Moges et al., demonstrated a strong correlation between congenital abnormalities and advanced maternal age (>35 years), with a 1.97-fold increased fetal risk. This was likely due to a higher incidence of chromosomal segregation errors [13]. Although ectrodactyly is not directly associated with gestational diabetes, oxidative stress from maternal metabolic disorders can interfere with embryonic morphogenesis [14]. Bellizzi et al. reported that maternal chronic hypertension elevates the risk of renal, limb, and orofacial congenital malformations [15]. In our case, a prenatal screening performed using 2D ultrasonography revealed no anomaly, which is in line with the findings by Singh et al. [9]. On the other hand, Bailess reported a case in which an isolated left-hand ectrodactyly was recognized by a routine 20-week fetal anomaly scan [16]. This missed diagnosis highlights the necessity for improved training in fetal limb evaluation and the use of 3D ultrasonography, particularly in resource-limited settings [9].

The management of ectrodactyly is tailored to each patient and depends on the severity of deformity, the degree of impairment, and cosmetic relevance [17]. The best outcomes are achieved through a multidisciplinary approach, involving orthopedic surgery, plastic surgery, and physiotherapy, to maximize limb functionality and aesthetic restoration [9].

## Conclusions

This appears to be the first documented case of ectrodactyly in Ghana. Ectrodactyly is detectable using 2D ultrasonography. This case highlights gaps in prenatal detection and congenital anomaly surveillance, particularly of limb defects in Ghana. Accurate reporting is necessary to determine actual burden estimates of congenital abnormalities in the country and guide resource allocation.
